# The computations that support simple decision-making: A comparison between the diffusion and urgency-gating models

**DOI:** 10.1038/s41598-017-16694-7

**Published:** 2017-11-27

**Authors:** Nathan J. Evans, Guy E. Hawkins, Udo Boehm, Eric-Jan Wagenmakers, Scott D. Brown

**Affiliations:** 1 Department of Psychology, Vanderbilt University, Nashville, USA; 2 School of Psychology, University of Newcastle, Callaghan, Australia; 3 Department of Experimental Psychology, University of Groningen, Groningen, The Netherlands; 4 Department of Psychology, University of Amsterdam, Amsterdam, The Netherlands

## Abstract

We investigate a question relevant to the psychology and neuroscience of perceptual decision-making: whether decisions are based on steadily accumulating evidence, or only on the most recent evidence. We report an empirical comparison between two of the most prominent examples of these theoretical positions, the diffusion model and the urgency-gating model, via model-based qualitative and quantitative comparisons. Our findings support the predictions of the diffusion model over the urgency-gating model, and therefore, the notion that evidence accumulates without much decay. Gross qualitative patterns and fine structural details of the data are inconsistent with the notion that decisions are based only on the most recent evidence. More generally, we discuss some strengths and weaknesses of scientific methods that investigate quantitative models by distilling the formal models to qualitative predictions.

## Introduction

For humans and many other animals, life presents a constant stream of decisions about basic survival, navigation, social interaction, and a host of other situations. For more than 50 years, a simple idea has provided the foundation of the study of decision making in neuroscience, psychology, cognitive science, and economics: the idea of “evidence accumulation”. Mathematical theories known as evidence accumulation models (EAMs) assume that decisions are made by gradually accumulating evidence from the environment in favor of each possible alternative. The first alternative to accumulate a threshold amount of evidence is selected for action. Through variations in this basic theme, accumulator models of decision making have helped explain cognitive and neurophysiological aspects of decision making^1–6^, and provided powerful ways to understand hundreds of important applied problems, from clinical disorders^7^ and personality^8^, to alcohol intoxication^9^, and sleep deprivation^10^. For modern reviews of EAMs and associated research in neuroscience, clinical, and other applied paradigms, see refs^11,12^.

The most commonly-studied EAM for choices between two alternatives is the diffusion model^2,3,13^, which proposes that the evidence accumulation process occurs as Brownian motion. The motion takes place between two absorbing boundaries, which represent the two response alternatives. The diffusion model thus assumes that evidence – which is normally distributed from moment to moment – is accumulated in a running total, and that a decision is triggered as soon as the total reaches a criterion. Apart from the diffusion model, there are many other EAMs which share this common structure^1,13–20^.

Recently, a new decision making model was proposed, the Urgency-Gating Model (UGM)^21–23^. The UGM made the intriguing proposal that the apparent steady accumulation of evidence is actually instead caused by *“time-varying gain, not by temporal integration”* ^22^, [p.11561]. The new idea of the UGM was that evidence is barely accumulated at all. Instead, moment-by-moment evidence samples are tracked by a rapidly-leaking evidence accumulator – a low-pass filter with a time constant of a few hundred milliseconds. These filtered samples are monitored until they exceed a threshold. To avoid waiting too long, the incoming samples are multiplied by an ever-increasing gain function which represents an internal “urgency signal”.

Evidence accumulation models that include leakage processes have been investigated previously. Early results^17,24^ were mixed, including some evidence for leaky evidence accumulation in decision-making paradigms involving slower decisions about time-varying environments. The qualitative pattern used to infer this support related to the differential effects of information provided early in the decision-making process vs. late in the process (though some comparisons found no evidence of leakage, e.g.^25^). These investigations have been based on a key intuition about the models, which leads to a strong qualitative prediction. All else being equal, leaky evidence accumulation implies that evidence which arrives early will have less influence on the decision than evidence which arrives late, because the early evidence has more time to “leak away”.

The “all else being equal” qualifier is important, because models that include leaky evidence accumulation and those that do not almost always differ from one another in more ways than just leakage. For example, the diffusion model and the UGM differ from each other in many ways. Firstly, the diffusion model assumes leak-free evidence accumulation, while the UGM assumes leaky accumulation. Secondly, the diffusion model assumes a time-homogeneous evidence accumulation process whereas the UGM assumes a time-inhomogeneous evidence accumulation process. That is, in the diffusion model the same rules govern evidence accumulation throughout each decision. In the UGM, there is a growing “urgency signal”, which changes the way accumulated evidence is used as the decision unfolds. There are still other differences between the models that can be important, and which change the models’ predictions (e.g. assumptions about decision-to-decision variability in model parameters).

When decisions are made about stimuli that do not vary with time, the limited investigation that has been conducted to date has shown that the diffusion model provides a better description of human data than the UGM, although the reverse may be true for data from monkeys^26^. The situation is less clear for decisions about stimuli in which the evidence is constantly varying. In such decisions, these two quantitative models have mostly been compared by distilling from them qualitative predictions, which are easy to test in data. Studies which have investigated decision stimuli in which early evidence and late evidence can sometimes favor different response options have mostly concluded in favor of the UGM, because they observed evidence in favor of leaky accumulation^21–23^. Another study with time-varying stimuli^27^ found the opposite – that early evidence influenced decisions, which is consistent with leak-free integration. However, this finding was inconclusive, because, for short decision times, the rapidly-leaking accumulation process of the UGM means that not all of the early evidence will have leaked away by the time a decision is made^28^. This work highlights two important points. Firstly, that slower decisions with longer response times may better qualitatively discriminate between the models, because the rapidly-leaking evidence accumulation process of the UGM will make different predictions than leak-free accumulation, over longer time scales. Secondly, that the “all else being equal” caveat is crucial in this debate: the psychological theories being contrasted differ in many important ways beyond the leakiness of evidence accumulation, and these extra differences have the potential to obscure or even reverse the qualitative model predictions that one might otherwise intuit.

**Figure 1:**
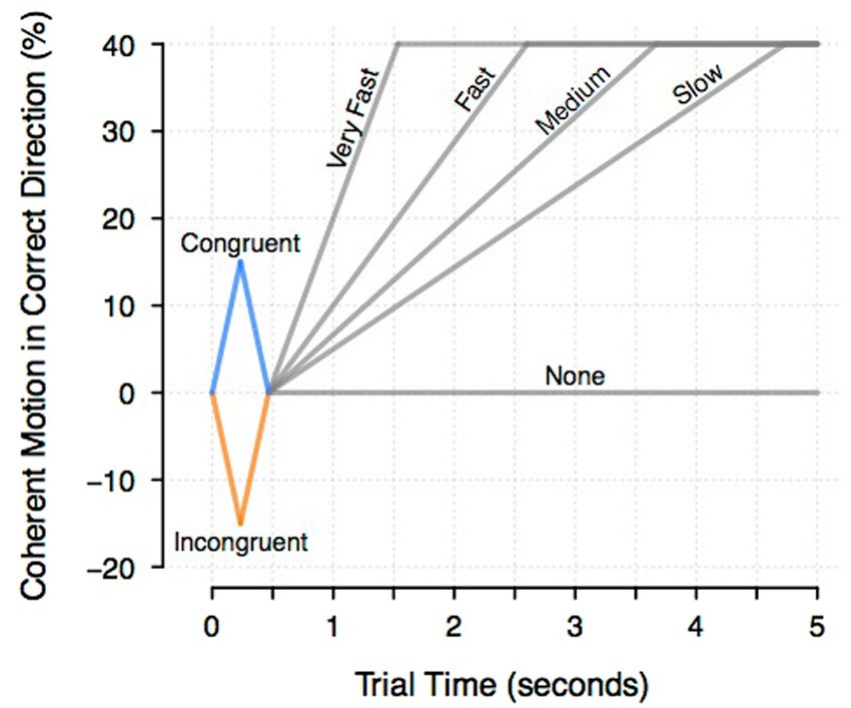
Illustration of the experiment design. The strength of evidence in the stimulus was manipulated by changing the coherence of the random dot motion (*y*-axis). The figure shows how strength of evidence changed over time within each decision trial (*x*-axis). Blue and orange lines show early evidence that is congruent and incongruent with late evidence, respectively. Following the presentation of early evidence, the congruent and incongruent conditions have the same rate of increase in late evidence, shown in gray lines, which differed across five levels.

To prevent mimicry issues seen in previous studies in the assessment of the qualitative trends predicted by the models, our study generates decision times which are substantially longer than the time constant of the evidence accumulation process in the UGM. We had people make decisions in which the early evidence sometimes differed from the late evidence, and in which decision times were systematically manipulated over a large range (Fig. 1 illustrates our design). Some decision trials had early evidence that was “congruent” in the sense that it favored the same response as the later evidence, and other decision trials had “incongruent” early evidence. We manipulated decision times by changing the rate at which late evidence arrived. All else being equal, leaky accumulation models, including the UGM, predict decreasing effects of the early evidence with slower decisions, because there is more time for the early evidence to leak away. By contrast, perfect integrators, including the diffusion model, predict that the effects of early information will remain constant across changes in decision time. (Note that these predictions are based on the potentially naive assumption that there is an unchanging drift rate – for the UGM, this would be the evidence multiplier – over the course of the entire trial. This has been a constant assumption within this debate; however, a non-stationary drift rate could potentially lead to different qualitative predictions.)

Additionally, previous investigations have sometimes examined only mean decision times. This can be an important limitation because it is often easy to specify a model which fits the mean decision time, if that model is not also constrained to predict the characteristic shape of the decision time distribution (e.g. see Luce’s classic textbook^29^ on this subject). Analyses of the joint distribution over response time and accuracy provide greater model constraint, and may better discriminate between the UGM and diffusion model.

## Results

We had 70 people each make decisions about the direction of motion in a random dot kinematogram (RDK)^30,31^. Half of the decision stimuli included a brief pulse of motion in the opposite direction to the ongoing motion (“incongruent” trials) and the other half of trials included a brief pulse in the same direction as the ongoing motion (“congruent” trials). The ongoing motion coherence in each trial increased at one of five “ramp rates”: none, slow, medium, fast, and very fast.

### Testing Qualitative Predictions from the Quantitative Models

**Figure 2:**
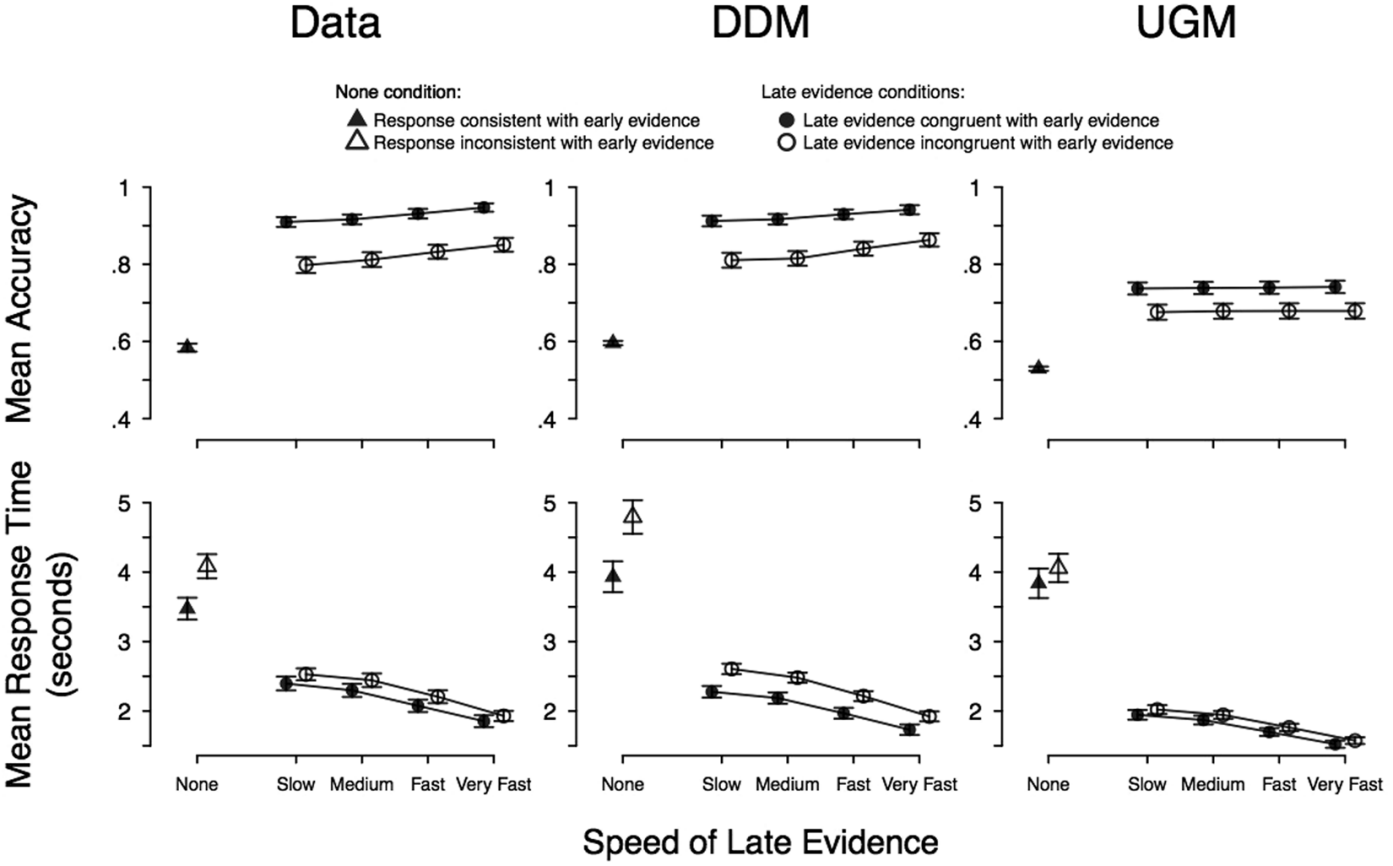
Mean accuracy (upper) and mean response time (lower) for congruent and incongruent conditions (filled and unfilled circles, respectively) as a function of the rate of change in evidence (*x*-axis). The errors bars display the between-subjects standard error of the mean. The left panels display results for the empirical data. Slower onset rates of late evidence led to lower accuracy and slower mean response times, though the differences between congruent and incongruent trials remained stable across onset rates. The middle panel and right panel display the predictions of the diffusion (DDM) and urgency-gating model (UGM) for these data trends, respectively. As can be seen, the diffusion model’s predictions match the effects seen in the data, with a difference between congruent and incongruent trials in accuracy and response time, whereas the UGM fails to match the data with almost no predicted differences. However, it should be noted that the diffusion does seem to over-predict the quantitative amount of difference seen between congruent and incongruent trials in mean response time, though the data does show evidence for a difference between these types of trials. For the condition with no late evidence, the diffusion provides a better account of the number of responses consistent with early evidence than the UGM, with the UGM under-predicting the amount of consistent responses. For the response times in the no late evidence condition, the diffusion over-predicts the difference between consistent and inconsistent responses, with the UGM providing a more accurate prediction of these times, although it under-predicts the difference.

Firstly, we contrasted the models using the qualitative predictions that have previously been ascribed to these two models. These qualitative predictions concern the mean response times and accuracy predicted by the models across the different conditions. Mean response times and accuracy from our data are shown in Fig. 2. A default Bayesian ANOVA^32^ (i.e., using default priors) on mean response time found the model that included both main effects of congruency and ramp rate, but no interaction between them, to provide the best marginal likelihood. Specifically, the analysis showed strong evidence for an increase in mean RT as the ramp rate became slower (Bayes factor of more than 10^80^-to-1 in favor of the alternative), which supports the effectiveness of our manipulation in generating different response times over ramp rate. Additionally, the analysis showed strong evidence that the difference in mean RT between congruent and incongruent trials did not differ over ramp rate (i.e., the congruency by ramp rate interaction; Bayes factor of 19-to-1 in favor of the null), which is consistent with the predictions attributed to the diffusion model. A default Bayesian ANOVA on accuracy again found the model that included both main effects of congruency and ramp rate, but no interaction between them, to provide the best marginal likelihood. Specifically, the analysis showed strong evidence that the difference in accuracy between congruent and incongruent trials did not differ over ramp rate (i.e., the congruency by ramp rate interaction; Bayes factor of 39-to-1 in favor of the null), which is again consistent with the predictions attributed to the diffusion model.

Note that we did not include the “none” ramp rate in these ANOVA analyses, because there is no objective definition of “congruent” and “incongruent” stimuli in these conditions, and what serves as a “correct” response in this condition is different from the way it is defined in the other four ramp rates. We instead display whether the responses were “consistent” or “inconsistent” with the early evidence presented. Thus, Fig. 2 shows a bias for responses to align with the early evidence in the “none” condition: more responses were consistent than inconsistent (Bayes factor of more than 10^8^-to-1). The consistent responses were also faster than inconsistent responses (Bayes factor of more than 10^8^-to-1). Qualitatively, these findings are consistent with the predictions of leak-free evidence accumulation models, like the diffusion model, because even very slow decisions showed an effect of early information. This is less consistent with models which assume leaky evidence accumulation, including the urgency gating model.

Next, we attempted to assess how long the effects of early evidence persisted over time, by looking at the differences in accuracy between congruent and incongruent trials across different intervals of the response time distributions. These comparisons are also known as conditional accuracy functions (CAFs), and have previously been used to assess whether evidence accumulation is leaky^33,34^.

**Figure 3:**
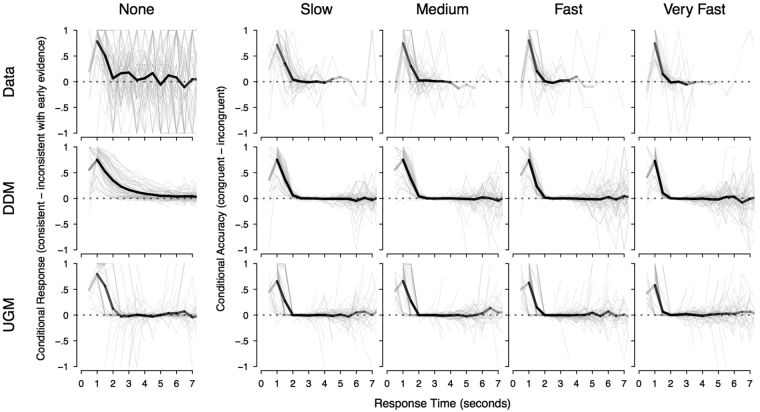
Conditional accuracy functions (CAFs), which take the difference in accuracy between congruent and incongruent trials across different bins of the response time distribution. The columns show different ramp rates. For the first column, the none condition, where there was no objective definition of congruent and incongruent, we took the difference between the proportion of consistent and inconsistent responses. The rows display the results of the empirical data (top row), and the predictions for these trends for the diffusion model (DDM; middle row) and the urgency-gating model (UGM; bottom row). Within each figure, the thick black line displays the group average and the thin gray lines show each participant. The transparency of the black line also indicates the number of subjects with responses in that interval, with more transparency (i.e., lighter colour) indicating fewer participants made responses in that interval. In general, the data displays a clear decreasing CAF, with all non-zero ramp rates converging to no difference between congruent and incongruent at around 2 s, and the none condition coming close to no difference between consistent and inconsistent responses at 2 s, and then displaying an inconsistent increasing and decreasing trend for still slower responses. The predictions of both models match these trends quite well. In the conditions with non-zero ramp rate, the predictions of the models are near indistinguishable from each other, and from the data. In the none condition, both models miss the trends somewhat, but in different ways. The UGM better predicts the initial decay, but incorrectly predicts that a very small proportion of participants respond in the early intervals, and that the trend flattens after the initial decay. The diffusion model successfully predicts that a large number of participants respond in the early intervals, and that the trend does not completely flatten after 2 s. However, it incorrectly predicts the rate of initial decay, with a smoothly decaying function opposed to the inconsistent one in the empirical data.

We calculated CAFs separately for each of the five ramp rate conditions, taking the difference between congruent and incongruent trials for the ramp rates with objectively correct responses, and the difference between consistent and inconsistent responses for the “none” ramp rate. The top row of Fig. 3 shows these CAFs both as group averages (black lines) and for every individual participant (gray lines). Overall, for the ramp rates with an objective definition of congruency, with increasing decision time there is a decreasing impact of the early evidence; the greater accuracy seen in congruent trials over incongruent trials disappears at around the 2 s mark. A similar, though more slowly decaying and less consistent, trend is present when observing the proportion of responses consistent with early evidence in the “none” ramp rate. However, in all plots, the trends become less clear as the intervals extend beyond 2 s, where the data are sparse. This is a difficulty of the CAF analysis technique: graphs in the slowest bins are dominated by a small subset of participants who had very slow response times. Corresponding problems have seen the use of the related “hazard function” analysis of response times discontinued^29^.

Interestingly, the two methods of qualitative analysis support opposite conclusions. The first method, which tests predictions for mean RT and accuracy, suggested that evidence accumulation is not subject to fast leakage. The second method, using CAF plots, suggested that there is leakage in evidence accumulation with a time course on the order of 2 seconds.

### Testing Quantitative Predictions from the Quantitative Models

A difficulty with testing qualitative predictions from quantitative models is that the predictions are usually generated by intuition of the researchers, based on their expectations about key components of the models operating in isolation. This entails the “all else being equal” assumption mentioned earlier, and as we show below, can lead to incorrect inferences being made. Given the obvious danger in intuiting qualitative predictions from complex models, we assessed the legitimacy of the qualitative predictions by generating actual quantitative predictions from the models. This serves as a check on whether the previously-reported tests of the qualitative predictions are actually relevant to the models being investigated. Following this, we directly contrast the models in their quantitative ability to fit the data.

#### Assessing the accuracy of the Qualitative Predictions

We assessed the accuracy of the previous qualitative predictions by examining the precise quantitative predictions made by these models. Using parameters estimated from fitting the models to the entire response time distributions, we generated synthetic data from the models, and replicated the analyses presented in the previous section on Qualitative Predictions.

For analyses of the mean response time and accuracy of each condition, the synthetic data generated qualitative patterns that agreed with our inferences from the predictions tested above: that the diffusion model provides a superior account of the data. As can be seen in Fig. 2, synthetic data from the diffusion model predict a sizeable difference between the congruent and incongruent conditions for both mean response time and accuracy, with this difference remaining fairly stable across ramp rates. Synthetic data from the UGM, on the other hand, show a very small difference in mean response time between congruent and incongruent trials, and a much smaller difference in accuracy than the diffusion model. In agreement with the analyses of the previous section, the predictions of the UGM do not match the data very well, underestimating accuracy, as well as the differences between accuracy and mean response time in the congruent and incongruent trials.

For decisions with no late evidence (the “none” condition), the UGM and diffusion model match the accuracy and response time data in opposite ways. For the observed response times, the UGM provides a better account than the diffusion model, but for the proportion of each response kind, the diffusion model provides a good fit while the UGM does not. Although the diffusion model perfectly matches the proportion of consistent responses, with the UGM under-predicting this amount, the diffusion predicts a longer mean response time for inconsistent trials than that observed in the empirical data, resulting in an over-prediction of the difference between the consistent and inconsistent response times. The UGM, on the other hand, provides an under-prediction of this difference, though it better matches the actual response times observed for the inconsistent trials.

What is surprising here is that the intuitive predictions made for each model were not always consistent with the models’ actual predictions. The diffusion model’s actual predictions matched the intuitive predictions for the model: no interaction for either mean response time or accuracy. On the other hand, the simulated data from the UGM also showed no interaction for these variables, which is opposite to the predictions previously ascribed (by intuition) to leaky models like the UGM. This suggests that the qualitative tests may be of limited use.

Next, we performed the corresponding analyses for the qualitative predictions about CAFs. The middle and bottom panels of Fig. 3 show CAF plots generated using synthetic data from the diffusion model and UGM, respectively. Both models predict an initial difference between congruent and incongruent trials, and both models also predict a gradual decrease as response times become longer, to the point where the differences have disappeared. It is clear from this analysis that the standard, intuitive predictions for CAFs attributed to the models are incorrect, and both models predict decreasing CAFs, just like those observed for the data.

**Figure 4:**
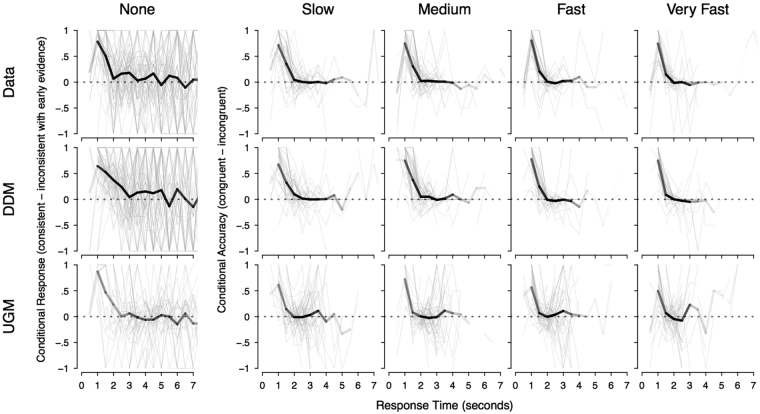
Conditional accuracy functions (CAFs), which are identical to Fig. 3, except that the model predictions for the diffusion model (DDM) and the urgency gating model (UGM) are now generated with a number of trials that matches those of the empirical data.

The initial proposal for using CAFs to discriminate between the two models relied on a clear qualitative difference: a decreasing CAF for the UGM, and a non-decreasing CAF for the diffusion model. Given that these intuitive predictions do not correspond to the actual predictions of the models, we explored whether some more subtle difference between the models’ actual predictions could be identified. If we restrict attention to just the “none” condition of Fig. 3, the UGM predicts a slightly faster rate of decline in the CAF than the diffusion model. Even in this condition, each model matches the data better than the other in different respects. In the fastest time bins (1–1.5 seconds), both models provide an accurate account of the initial decay, though the UGM under-predicts the proportion of subjects who made responses in these bins. In the 2 second bin, the data are much better explained by the UGM, with a sharp decline to almost zero. However, immediately after this in the slower time bins, the CAF data are more consistent with the diffusion model. It is quite possible that these fluctuating effects are attributable to the finite sample size. This hypothesis is supported by the analyses shown in Fig. 4, which illustrates the two models’ predictions for the CAFs, when the models are used to simulate the same size data samples as our experiment. With that sample size, the models’ predictions for this particular comparison are both quite noisy, making it very difficult to be confident which model best matches the CAFs.

The most important conclusion from these analyses is that the previous finding – that CAFs decrease with time – does not discriminate between the UGM and diffusion model. This is because that qualitative prediction is based on an incorrect intuition, and the noise inherent in standard data sets precludes decisive conclusions. In each CAF, as we move from left to right, the plot is increasingly dominated by the slowest responses from the slowest participants. This makes it difficult to know how much weight to ascribe to discrepancies between the models and the data in these plots.

#### Comparison of the quantitative fits to the full choice response time distributions

**Figure 5:**
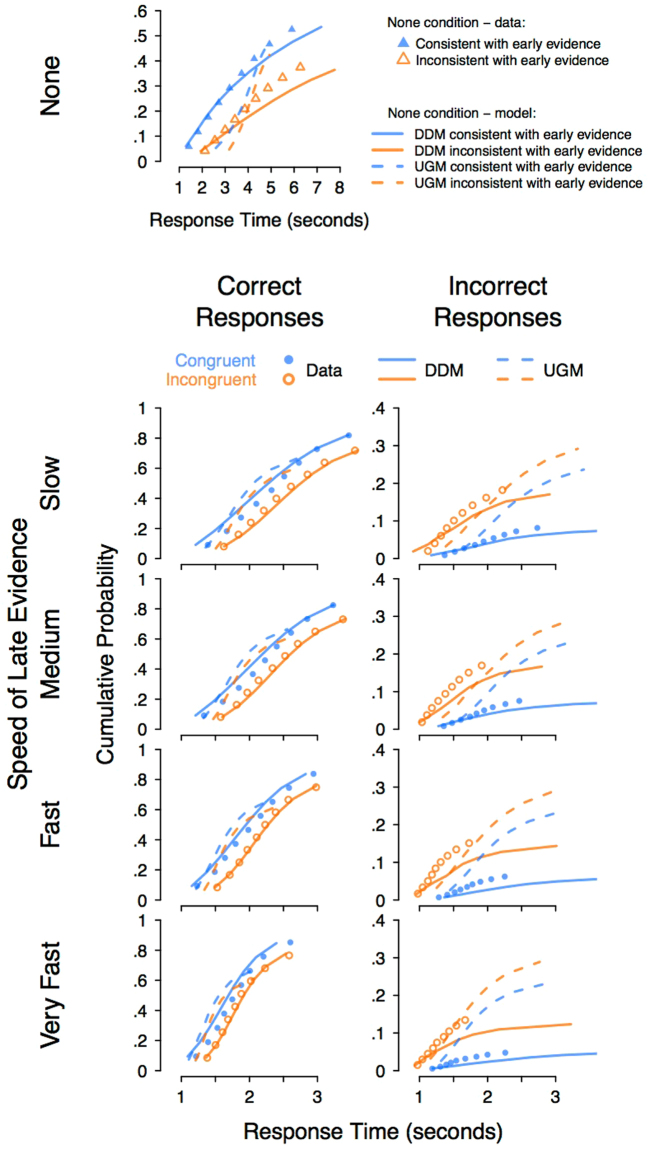
Cumulative distribution function (CDF) plots of the quantitative fit of the UGM and diffusion model (DDM) to the response time distributions, averaged over participants. The *y*-axis displays the cumulative probability of a response and the *x*-axis displays the response time. Dots show the data and lines show the predictions of the DDM (solid lines) and UGM (dashed lines). Blue and orange symbols represent the congruent and incongruent conditions, respectively. Distributions of correct and incorrect responses are shown in the left and right columns, respectively. For the condition with no late evidence presented – the top panels – data and model predictions are shown in terms of responses consistent or inconsistent with the late evidence. The DDM provides a better account of the key trends in most conditions, although the UGM provides a better fit to the very fastest quantiles for the congruent trials with late evidence presented. Note: The *y*-axis scaling differs across panels, to accommodate the different response proportions over correct and incorrect responses. The *x*-axis scaling of the top row is different from the other rows, due to the elongation of the response time distribution in the condition with no late evidence.

Next, we compared the models quantitatively, by fitting them to the joint distributions of response time and accuracy across all conditions. We used the data to estimate the parameters of the diffusion model and the UGM, separately for each participant. We then constructed joint cumulative distribution functions (CDFs) over correct and incorrect response time distributions, for each participant and each condition, and averaged these. Averaging joint CDFs across subjects does not have the same statistical problems as averaging other measures of distributions, such as histograms. As above, we again use the terms “consistent” and “inconsistent” for the “none” condition. The data and corresponding model predictions are shown in Fig. 5.

The diffusion model provides a better fit to the data than the UGM. For example, in the conditions with late evidence presented, on average the diffusion model underestimates the median correct RT by 27ms, and the accuracy by 0.4%, whereas the UGM underestimates median RT by 445ms and accuracy by 16%. This pattern occurs throughout nearly the entirety of the correct response time distributions, with the diffusion model better predicting every quantile of the correct response time distribution for incongruent trials, and better predicting the majority of quantiles for congruent trials. However, for the congruent trials with late evidence presented, the UGM provides a better fit than the diffusion model to the very fastest responses, corresponding to the leading edge of the RT distribution. This benefit applies mostly to the 10^*th*^ and 20^*th*^ percentiles, and suggests that the UGM may be more accurately capturing what happens in those few trials where very fast decisions are made, on easy stimuli especially. In addition, although the diffusion model provides a better account of the incorrect responses than the UGM, both models substantially misfit these data.

**Figure 6:**
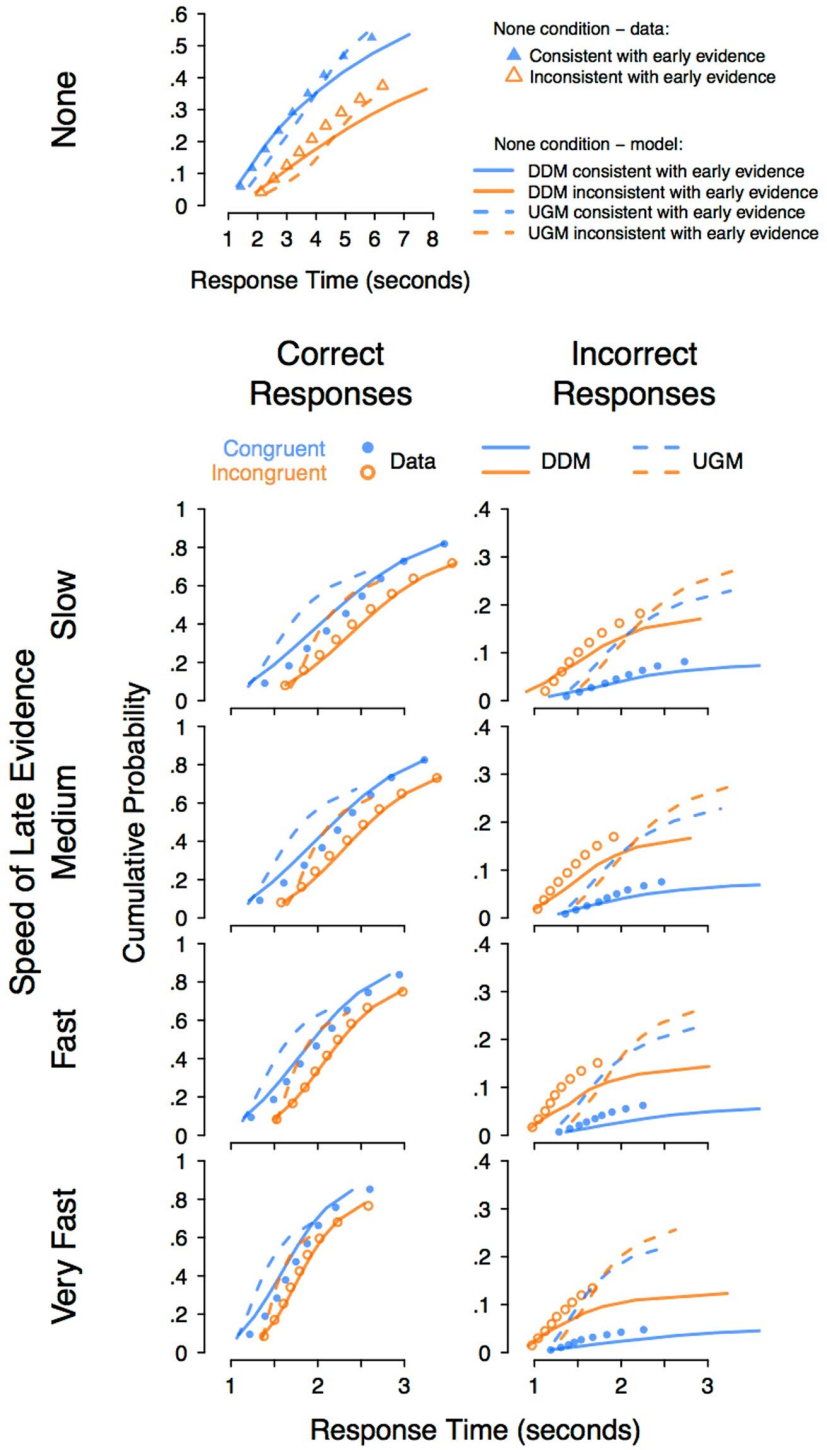
Cumulative distribution function (CDF) plots of the quantitative fit of the UGM, with the time constant allowed to vary as a free parameter, and diffusion model (DDM) to the response time distributions, averaged over participants. All other details are identical to Fig. 5. Despite the additional freedom of the free time constant, the UGM still fails to capture the elongation of the correct response time distributions, or the overall error rate.

There appear to be two main reasons why the UGM does not fit the data well. Firstly, the UGM wrongly predicts a very small difference between congruent and incongruent trials. The data, and the diffusion model, show moderate to large effects of congruency, on both accuracy and RT. It is possible that this problem may be alleviated by allowing the UGM’s low-pass filter to have a very long time constant, making its evidence accumulation process less leaky, and more like the diffusion model. We explored this possibility by fitting the UGM with the time constant as a free parameter. This addition improved the UGM’s ability to account for the data, although the resulting fit was still worse than the diffusion model’s fit (see Fig. 6).

When looking at predictions from the “none” condition, the diffusion model continues to perform much better than the UGM, with the UGM providing an extremely poor fit to the data. However, the diffusion also provides some fairly large misfit on the later quantiles, meaning that neither model provides a great fit to the “none” condition at an absolute level. When looking at the fits of the UGM with a free time constant to the “none” condition, the fits are much better than the regular UGM, and are equal to, if not better than, the diffusion. Although these the fits are about equally good for the diffusion model and the UGM with a free time constant, both models appear to misfit in quite different ways: the UGM over-predicts the early quantiles and provides a very tight fit to the late quantiles, suggesting a truncation of the distribution relative to the empirical data, whereas the diffusion model predicts the opposite, with a tight fit to the early quantiles, and an over-prediction of the late quantiles.

The second reason why the UGM does not fit the data is that it fails to predict the shape of response time distributions (see also UGM fits from^26^). The most notable issue in our data is that the UGM cannot correctly predict the variance of the distribution, or the proportion of correct responses. This is evident in Fig. 5 in which the predicted CDFs from the UGM (dashed lines) all greatly under-predict the width of the response time distribution, as well as the proportion of correct responses. This problem arises because of the second major difference between the UGM and diffusion models: the UGM’s urgency signal. The increasing urgency signal in the UGM results in ever-increasing variance of the moment-by-moment evidence. As the late portions of accumulation are dominated by the noise process, slower responses are very unlikely, and responses become more random and less accurate. This finding of our study highlights the importance of using quantitative modeling, as the failure of the UGM to capture the shape of the distribution would be missed in a qualitative comparison against mean RT alone.

## Discussion

Our study contributes to the debate about the role of evidence accumulation in decision making, and further adds to knowledge about the different ways in which this debate might be advanced. We contrasted two prominent models, the diffusion model and the urgency-gating model, by fitting them to full response time distributions, and comparing quantitative model misfit. We also more deeply explored previous model evaluation methods based on qualitative predictions for the quantitative models. Our analyses of qualitative predictions revealed that certain previous predictions had been incorrectly attributed to the models. When these errors were corrected, the qualitative model tests confirmed the quantitative analyses: finding evidence in favor of leak-free evidence accumulation.

This correction centered on the analysis of conditional accuracy functions (CAFs), which have been used in previous studies that have attempted to contrast models with and without leakage^33,34^. The naive intuition about CAFs is that leaky evidence accumulation should predict a CAF that decreases with response time, in contrast to leak-free evidence accumulation, which should predict a CAF that remains constant with response time. However, quantitative analyses of the models demonstrated that these naive predictions are incorrect – both models predict a decreasing CAF. This has previously been noted, as a consequence of assuming between-trial variability in model parameters^35^. Variability causes speed and accuracy to covary due to samples from the higher points of the drift rate distribution being both faster and more accurate, and samples from the lower points of the drift rate distribution being both slower and less accurate^3^. Our finding underscores the difficulties that arise when testing quantitative models via distilling qualitative predictions from them. Related difficulties have previously beset many other similar attempts, with naive predictions turning out to be wrong, when reasonable other model assumptions are made. For example, the extensive debates about whether curvilinear zROC slopes falsify different recognition memory models. These debates remain ongoing primarily because the naive predictions from the models are heavily influenced by other aspects of the models than those being purportedly tested. Quantitative model selection methods avoid these problems by directly examining the predictions from the full, as-specified, models.

Another unhelpful side-effect of using qualitative predictions to test quantitative models is the restricted focus used within the assessment, where the comparison between the models is based upon a single aspect of the data – sometimes just a few data points. This restricted focus is necessary to sufficiently simplify the model comparison to a qualitative level, for example, by restricting analyses to only mean response time and mean accuracy, or only to one condition or another of an experiment. We fit the models directly to the full data set: the distribution of response times jointly over correct and incorrect responses. Fitting the models in this way allows them to be clearly distinguished, as they make very different predictions for response time distributions (see Fig. 5).

Although the UGM provided a poor quantitative fit to the data in this study, there may always exist adjustments or extensions to the model which could allow it to better account for the data. One possible adjustment would be to allow the urgency signal to change across the different ramp rates, which could mitigate the variance inflation issue. However, this is theoretically problematic because, to successfully fit the data, the urgency signal would need to behave opposite to its definition. The urgency signal is a mechanism that stops people from spending too much time on decisions, by increasing urgency for longer decision times. However, to successfully fit our data, a *smaller* signal would be needed in the slower ramp rates, and a larger signal would be needed in the faster ramp rates. This would have the effect of increasing urgency for *short* decisions and decreasing urgency for *long* decisions. We did not pursue this model extension, as it seems to violate the theoretical underpinnings of the UGM. However, it should be noted that recent research in paradigms that somewhat resemble our differing ramp rates has found that faster ramp rates produce higher neural spikes^36^. Although there are several explanations for this finding, this research falls in line with what would be expected from an UGM with a stronger urgency signal for faster ramp rates, and might be an interesting avenue for future research.

Evidence accumulation models have successfully explained decision-making data in fields as diverse as economics, psychology, and neuroscience. Our study compared the most popular evidence accumulation model, the diffusion model, to the urgency-gating model, which assumes fast leakage of evidence during the accumulation process. Contrary to the predictions of the urgency-gating model, our study found that information provided early in the decision-making process had a lasting and relatively constant influence on the decision outcome. Notwithstanding some nuances, our results provide evidence in favor of leak-free evidence accumulation during decision making.

## Methods

### Participants

The experimental protocols were approved by the University of Newcastle Human Research Ethics Committee, and our methods were carried out in accordance to their guidelines and regulations, which included obtaining informed consent from all participants. After data collection, five participants were excluded due to low accuracy (less than 60%).

### Design, Task and Procedure

We treated our study as a 5 (evidence ramp rate; none, slow, medium, fast, very fast) by 2 (congruency; congruent, incongruent) within-subjects design. The congruency manipulation referred to whether the evidence presented early in each trial was congruent or incongruent to the evidence presented late in the trial, where the late evidence determined the correct answer. The ramp rate referred to the rate that the late evidence was gradually introduced in each trial (exact details below), with “none” representing no late evidence being presented. For the “none” condition, there was no way of defining the congruency of a trial or the correctness of responses based on our previous definitions, as there was no late evidence presented. Instead, we instead defined the responses as either “consistent” or “inconsistent” with the early evidence presented within this condition.

We used a random dot kinematogram (RDK) with the white noise algorithm^30^. In each trial, 40 white dots (3 pixel diameter each) were shown inside a 150-pixel diameter circle in the center of a black screen. On each video frame (66.7 ms), a portion of the dots were randomly selected to move coherently toward the top left or top right of the screen. These dots moved 3 pixels up and 3 pixels left/right on each frame, where the number of coherently moving dots was determined by the ramp rate and the elapsed time in the current trial. All other dots were randomly replaced within the area. Participants were asked to decide whether the overall pattern of dots was moving towards the top-left or the top-right of the screen, using the “z” and “/” keys, respectively, with the correct answer relating to the direction of the late evidence. (Note that fewer than 0.7% of included decisions were made during the period that would have resulted in the current evidence on screen being in the opposite direction to the late evidence, with 43/65 included participants having 0%.)

Before each trial, participants were presented with a fixation cross in the center of the screen. This was shown for a random amount of time drawn from a truncated exponential distribution with mean 700 ms, truncated at 4,800 ms, with a 200 ms offset added. After each trial, participants received feedback on their performance for 300 ms, with an additional error timeout of 500 ms for incorrect responses (defined relative to the late evidence) followed by a 100 ms blank screen. Within each block all conditions were randomly intermixed, with an equal number of trials from each cell of the design, and an equal number of trials with dots moving in each direction. Participants completed 8 blocks of 60 trials each, with the initial block treated as a practice block and removed from further analysis.

During the course of each trial, the number of dots moving coherently on any one frame was determined by the elapsed time in the trial, and the ramp rate of the trial. At the beginning of the trial, the coherence started at 0 dots, moved up to 6 dots (15% motion coherence), and then back down to 0 dots over the course of 7 frames (467 ms), in steps of 2 dots at a time. The direction of this early motion was determined by whether the trial was congruent or incongruent, with this referring to whether the direction of early evidence matched the direction of late evidence. After this early evidence, the late evidence was presented, which moved at a linear rate up to a maximum coherence of 16 dots (40%), where the number of frames per dot increase in coherence was determined by the ramp rate. These rates were every frame (66.7 ms; ‘very fast’), every 2 frames (133 ms; ‘fast’), every 3 frames (200 ms; ‘medium’), and every 4 frames (267 ms; ‘slow’). Additionally, there were some trials that presented no late evidence, where the trial coherence stayed at 0 once the early evidence had finished (‘none’). Figure 1 provides a schematic representation of the change in evidence for each ramp rate over the course of a trial.

### Data Analysis

Initial analyses focused on the gross measures of mean response time and accuracy. To compare the qualitative predictions of the UGM and diffusion model, we used a 2-way default Bayesian ANOVA^32^. The effect of interest was the two-way interaction between ramp rate and congruency, which is predicted by the UGM but not by standard evidence accumulation models. Specifically, as the time between early evidence and the decision increases (as we expect will result from the ramp rate becoming slower), the UGM predicts that early evidence will have a decreasing effect on decisions, whereas the diffusion model predicts the effect of early evidence to be constant.

The next analysis involved creating conditional accuracy functions (CAFs) that displayed the change in the difference in accuracy between congruent and incongruent trials across the response time distribution, for each ramp rate. This involved segmenting the response time distributions for each condition into intervals, with the intervals defined as 500 ms bins, starting at 0 ms and continuing through to 10,000 ms. Once the intervals were calculated, plots were made for the difference between congruent and incongruent trials in accuracy, for each of the intervals. For the “none” condition, this was instead done as the difference in the proportion of consistent and inconsistent responses. These were done both separately for each participant, as well as group-averaged.

The previous two qualitative analyses compared the empirical data to both the intuitive predictions of the models, as well as the quantitative predictions made by the models. The model predictions for each analysis were obtain through fitting the models to the response time distributions of the data, using the methods noted below for the quantitative model fitting. After this, model predictive data was generated, and qualitatively contrasted in the same manner as the empirical data.

Subsequent analyses compared the models’ predictions against the data in more detail. To ensure that the method we used was robust in optimizing the parameter values for each model, our fitting method followed that of previous studies that tested the diffusion model against the UGM and other time-inhomogeneous evidence accumulation models using extensive parameter recovery analyses^26,37^. We independently fit both models to each participant’s data. To identify the most likely set of parameters for each participant, the observed response time deciles for correct and error responses were calculated. For a given parameter set, the models make predictions for the number of responses expected in each decile-bin, and these predictions were compared to the observed counts. Parameters were optimized through QMPE^38^, and the parameter search was conducted with the differential evolution algorithm, via the R package “DEoptim”^39^. As no analytic solution exists thus far for the probability density function of the UGM, the predicted response time distributions for each set of parameters for each model were obtained through simulating 10,000 synthetic trials with each proposed parameter combination. The complete algorithms by which the models were simulated are provided in the section below (“Simulating the Models”).

Each model was defined by four free parameters, which by conventional standards suggests that the complexity of the models was relatively low, and approximately the same (though this is not always the case, see^40–42^). One common parameter between models was the non-decision time parameter, which governed the time involved in non-decision related processes such as stimulus encoding and response execution. Each model contained a threshold parameter, which represented the amount of evidence required to trigger a decision. For the diffusion model the threshold was parameterized as the distance from one threshold to the opposing threshold, and for the UGM the threshold was parameterized as the distance from one threshold to the middle of the two thresholds. The final two parameters governed the mean and standard deviation of the drift rate distribution for the two models, with a drift rate being randomly sampled from this distribution for each trial. In standard practice, this distribution would govern the rate of evidence accumulation for the diffusion model, and the amount of evidence at any point in time for the UGM. However, as our experiment contained constantly changing amounts of evidence, we used these parameters as scaling factors for the actual amount of evidence present at any point in time, with the diffusion model’s functional form accumulating this over the course of a trial, and the UGMs functional form updating this over the trial, at a rate governed by the low-pass filter time constant, which was fixed at 100 ms in the initial fits, and allowed to be a free parameter in the second set of fits. For the UGM, the urgency signal was fixed to scale linearly with time at a value of 1, as by convention^26^. For both models the starting point of evidence was fixed to be an equal distance from the two thresholds, reflecting no bias in the process. The starting point was also kept constant from trial-to-trial (i.e., no start point noise, as is commonly allowed in diffusion model fits^3^). The within-trial noise was fixed to be 0.1 for the diffusion model, and 100 for the UGM, following conventions^26^.

### Simulating the Models

The generative forms of the models used in this analysis were specified as stochastic differential equations, and simulated using Euler’s method^43^. To simulate a single decision for the diffusion model, an evidence counter was initialised to $x=\frac{a}{2}$, where *a* is a parameter for the separation between the response boundaries. This counter was iteratively updated according to:

1 $${x}_{t+{\rm{\Delta }}}\mapsto {x}_{t}+vC{\rm{\Delta }}+sw\sqrt{{\rm{\Delta }}}$$

Here, Δ is a simulation step size (we used 10 ms), *v* is a drift rate, *w* is an i.i.d. sample from a standard normal distribution, and *C* is the coherence of the random dot motion stimulus on the current time step of that simulated decision. Parameter *s* is a scaling parameter of the model and was fixed arbitrarily to *s* = 0.1. A simulated decision was made whenever *x* > *a* or *x* < 0, and the associated decision time was $t-\frac{{\rm{\Delta }}}{2}+{t}_{0}$, where *t* is the crossing time, and *t*_0_ is a parameter representing the time taken for processes outside of the decision itself, such as perception and response execution. The drift rate *v* was sampled from a normal distribution on each simulated decision, with standard deviation *η* (a free parameter) and mean given by *v*.

A similar algorithm was used to simulate a single decision for the UGM, but with two key differences: leaky integration, and an increasing urgency signal. To model leaky integration, we implemented a low-pass filter with time constant given by *τ*. Thus, the update equation for the evidence accumulator changed from diffusion model’s Equation 1 to:

2 $${x}_{t+{\rm{\Delta }}}\mapsto \frac{\tau }{\tau +{\rm{\Delta }}}{x}_{t}+\frac{{\rm{\Delta }}}{\tau +{\rm{\Delta }}}(vC{\rm{\Delta }}+sw\sqrt{{\rm{\Delta }}})$$

Quantities *v*, Δ, *s*, and *w* were defined exactly the same as for the diffusion model. To implement the urgency signal, the instantaneous value of the evidence accumulator (*x*_*t*_) was multiplied by the time measured in milliseconds since the onset of evidence accumulation (*t* − *t*_0_). This sets the scaling parameter for the urgency signal to a value of 1 *msec* ^−1^. This functional form also required the crossing of the threshold to occur at *x* > *a* or *x* < *−a*. The mean and standard deviation of the best fitting parameters values for the diffusion model, the standard UGM, and the UGM with a free time constant, can be seen in Table 1.

**Table 1:** The mean (M) and standard deviation (SD) across subjects for the best fitting parameter values for the diffusion model (Diffusion), the UGM (UGM), and the UGM with a free time constant (UGM_*free*_). For the UGM fits, the time constant was fixed at 100.

	*v*	*η*	*a*	*t*_*0*_	*τ*
Diffusion M	0.03	0.01	0.42	0.28	—
Diffusion SD	0.01	0.01	0.15	0.14	—
UGM M	7.05	11.13	53499	0.39	—
UGM SD	5.19	6.03	26034	0.28	—
UGM_*free*_ M	3.64	6.78	6655	0.22	4105
UGM_*free*_ SD	3.39	8.31	8539	0.22	2625

### Data Availability

The data used within this study, along with the experimental and analysis code, can all be found at: https://osf.io/a24h7/.
